# Life satisfaction two-years after stroke onset: the effects of gender, sex occupational status, memory function and quality of life among stroke patients (Newsqol) and their family caregivers (Whoqol-bref) in Luxembourg

**DOI:** 10.1186/1471-2377-12-105

**Published:** 2012-09-25

**Authors:** Michèle Baumann, Sophie Couffignal, Etienne Le Bihan, Nearkasen Chau

**Affiliations:** 1 Medical Sociology, scientific director of the project, INSIDE Research Unit, University of Luxembourg, L-7201, Walferdange, Luxembourg; 2 Epidemiology, Centre for Health Studies, Public Research Centre for Health, L-1445, Strassen, Luxembourg; 3 Statistician, INSIDE Research Unit, University of Luxembourg, L-7201, Walferdange, Luxembourg; 4 Epidemiology, INSERM, U669, Univ Paris-Sud and Univ Paris Descartes, UMR-S0669, Paris, France

**Keywords:** Cerebrovascular disease, Life satisfaction, Quality of life, Newsqol, Whoqol-bref, Post-stroke patients, Family caregivers

## Abstract

**Background:**

Life satisfaction (LS) of cerebrovascular disease survivors and their family caregivers may relate to socioeconomic factors, impaired functions, health-related quality of life (QoL), but their respective influences remain unclear. This study assessed, two years post-stroke onset, the effects of these factors on patients’ LS and family caregivers’ LS in Luxembourg.

**Methods:**

All stroke patients admitted to all hospitals in Luxembourg were identified by the *‘Inspection Général de la Sécurité Sociale*’ using the only national system database for care expenditure reimbursement. Their diagnosis was confirmed by medical investigator. The sample included ninety four patients living at home having given consent (mean age 65.5 years) and sixty two main caregivers (mean age 59.3 years). Questionnaires were completed during face-to-face interviews. LS was assessed via European single question (range 1–10), survivors’ QoL via Newsqol (11 dimensions), and caregivers’ QoL via Whoqol-bref (4 domains) (range 0–100). Data were analysed using multiple regression models.

**Results:**

Two years after stroke onset, 44.7% of patients suffered from impaired sensory function, 35.1% from impaired motor function, and 31.9% from impaired memory function. Mean patient’ LS was 7.1/10 (SD 1.9). It was higher in women (+12.4) and lower among unemployed socioeconomically active patients (−13.1, vs. retired people). Adjusted for sex, occupation, impaired motor and memory functions, LS positively correlated with scores of Newsqol feelings, sleep, emotion, cognition and pain dimensions (slopes 0.20 to 0.31), but did not correlate with those of caregivers’ Whoqol-bref domains. Family caregiver’ LS was 7.2 (SD 1.7). It was lower in those with patients suffering from impaired memory function (−12.8) as well as from feelings and emotion issues (slopes 0.22). It was associated with all caregivers’ Whoqol-bref domains (physical health, psychological health, environment, and social relationships) (slopes 0.53 to 0.68).

**Conclusions:**

Two-year post-cerebrovascular disease patient’ LS was associated with gender, occupation, and impaired memory function. It correlated with feelings, sleep, emotion, cognition, and pain issues. Family caregivers of patients with impaired memory function had lower LS. Family caregiver’ LS correlated with dimensions of patients’ feelings (less independent, yourself, life changed, depressed, useless, less control because of stroke) and emotion (get more emotional, fear of another stroke or to become dependent on others), and with their own QoL. LS, Newsqol, and Whoqol appeared to be appropriate tools. Our findings may be useful for policy makers in relation to family and medical-social issues of stroke home-based rehabilitation.

## Background

Cerebrovascular diseases are a high priority for European health and social policy makers due to their high prevalence and also to the fact that they cause long-term disabilities and substantial socioeconomic and emotional impacts on daily life [1]. In Luxembourg, they are the principal cause of acquired long-term disabilities and the third leading cause of death [2]. Stroke-related disabilities are not exclusively physical, but dysphasia and memory disorders can threaten person’ sense of self and have substantial socioeconomic, practical and emotional impacts [3]. The consequences of cerebrovascular disease constitute a challenge for individuals to maintain autonomy and life satisfaction (LS) which is a global perceptual measure of the degree of discrepancy between individual aspirations and achievements or contentment [4,5]. The Eurofund (the European Foundation for the Improvement of Living and Working Conditions, a European Union body working in specialised areas of European Union policy) committee considers the monitoring of the individual situation in the society and the social progress of Europeans to be a key element [5,6]. We opted for this approach among stoke patients and their family caregivers in the belief that QoL domains determine LS.

Because of long-term disabilities and high mortality risk over time [2] we need to monitor socioeconomic conditions and health-related issues and their effects on stoke survivors’ LS. Socioeconomic factors such as gender, age, education, and occupation are well-known potential risk factors for a wide range of health-related issues (substance use, mental difficulties, disability, etc.) and mortality [7]. Lower socioeconomic status was associated with a higher risk of stroke [8]. Health may also be altered by motor function problems, post-stroke psychological distress and restrictions in routine, leisure and work activities [9]. Cerebrovascular disease affects health in combination with social and material factors [10]. It may be postulated that the effect of stroke would be greater in families already facing disadvantages in healthcare, and that the effect of health problems will be greater when the individuals confront social disadvantage [11]. A study in the chronic phase found that 12 to 24 months after discharge from inpatient rehabilitation, LS decreased [12]. Another study showed that disability remained highly prevalent three years post-stroke [13]. Post-stroke psychological suffering is associated with impaired quality of life (QoL), functional limitations and healthcare use [14]. Among health-related issues, the knowledge of the respective roles of main stroke-related impaired functions (such as those concerning motor, visual, sensory, language, and memory functions) and QoL domains related to stroke may be useful for policy interventions aiming at improving health and daily life of patients. Indeed, patients may have to adjust their lifestyles, rearrange home, adapt communication styles, and revise plans for the future.

For stroke patients at home, family caregivers (spouses, partners, etc.) may play an important role in patients’ aid, care, and LS. Family caregiver’ LS is thus important. Cerebrovascular disease is an important family issue, particularly for spouses [15]. It affects not only the QoL and mental health of patients but also those of their close relatives [12,13,16-19]. Compared to controls, caregivers of stroke survivors commonly have lower QoL, higher prevalence of psychological distress, a greater economic burden, and an impoverishment of their social lives [20,21]. The “Helsingborg Declaration 2006 on European Stroke Strategies” highlights the importance of stroke management in several areas [22] in which family caregivers should play an essential role. In Sweden, an amendment in 2009 has recognised repercussions for informal caregivers, and recommends to minimise their physical and psychological strains and burden. But, caregiver strain and its consequence on its LS have remained an under-researched area [23]. Then, a critical question is whether and how patients’ socioeconomic characteristics and stroke-related impaired functions may impact family caregivers’ LS? Another pertinent question is whether among family caregivers, certain QoL domains may affect LS? The knowledge of these issues may identify patients and caregivers because they are now ‘two populations at risk’ for our social and public health system. Social and health-related aspects of QoL survivors and spouse are known to affect their LS, but their respective influence remains unclear [24].

An important problem concerns stroke-related QoL domains. In the literature, patient’ QoL has been assessed using various generic measures including health-related QoL, Sickness Impact Profile, and Nottingham Health Profile. Most of these measures fail to cover important stroke concerns such as communication, concentration, and memory. A recent review of the literature involving informal caregivers of stroke survivors (with and without aphasia) reported that all instruments used were generic [25]. Stroke-specific instruments available at the start of this study lack comprehensiveness, are not patient-centred and their validity and reliability remain unproved [26]. The Newsqol appears to be an appropriate stroke-related measure in terms of dimensions explored. But, no investigation has studied the relationship between LS and Newsqol [27] while it is of interest to know which Newsqol dimensions are affected by cerebrovascular disease and may influence LS.

The present study aimed at assessing, two years after cerebrovascular disease onset, effects on patients’ life satisfaction of socioeconomic factors, impaired functions (motor, visual, sensory, language, and memory), quality of life (via Newsqol which measure stroke-related QoL dimensions), and their family caregivers’ QoL (via Whoqol-bref which measures physical, psychological, environment, and social relationship domains) in Luxembourg. The survey further evaluated the effects of these factors on the family caregivers’ life satisfaction.

## Methods

### Study design, sample and recruitment

This was a retrospective health record audit involving all stroke survivors (797 patients) admitted to all hospitals in Luxembourg were identified in the *‘Inspection Générale de la Sécurité Sociale’* (the only national system for care expenditure reimbursement). The system database allowed us to first identify all treated stoke patients. Their status – living or deceased – was obtained from the Civil Status Registry.

#### Inclusion criteria

– Living in Luxembourg at cerebrovascular disease onset.

– Hospitalised in Luxembourg between 1^st^ July 2006 and 30^th^ June 2007.

– A clinically diagnosed stroke (hospital discharge code based on the International Statistical Classification of Diseases and Related Health Problems – 10^th^ revision (ICD-10 codes: I60, I61, I62, I63, I64, and G46)). Patients who had transitory ischemic attacks (TIAs) were excluded. Aphasic patients were not excluded because the researchers were trained to communicate with them. To interview aphasic patients or patients with troubles of elocution, researchers used a large laptop with a visual questionnaire. They could show it to the patients, and read the questions with them, and then the patients could answer with a pointer. They were also trained by a speech therapist to develop an empathetic and comprehensive attitude (as the time of these interviews was much longer).

– Resident in Luxembourg (not in an institution) two years after cerebrovascular disease.

– Understanding by patient or primary caregiver of one of the four languages (Luxembourgish, Portuguese, French and German) used for the face-to-face interview (1 case excluded).

– And valid addresses (11 cases excluded).

An informational letter and a request for written informed consent were sent to 374 retained patients to obtain their agreement to consult their hospitalisation and rehabilitation records, and to explain the aims of the national survey and to give their authorisation for a researcher to visit their home. Clinical diagnosis of cerebrovascular disease was confirmed by the medical investigator.

#### Characterization of the stroke severity, subtypes and risk factor profile of patients

The Barthel Index or modified Rankin Score scales were chosen by the expert neurologist on the investigative team as a measure of stroke severity. However, neither internationally recognized standardized scale was documented in the medical records reviewed. Thus, stroke severity was estimated based on the presence of clinical signs at the admission or at the time of maximum severity during the 1^st^ week, at the occurrence of an auricular fibrillation during the hospitalisation and a severe arterial hypertension to the admission [28].

### Sample

After receiving 102 signed informed consents (30 refusals and 242 missing answers) the research teams telephoned (up to five attempts) to make an appointment at the patient's home with the main caregiver identified by the patient as ‘*the person who mostly takes care of me since the cerebrovascular disease event*’.

The consents of the main caregivers were obtained at that visit. Two researchers, one per interview, conducted the face-to-face structured interviews supported by a questionnaire.

### Ethical restriction

The protocol was approved by the National Committee of Research Ethics (NCRE) and notified to the Committee for Data Protection of Luxembourg. Although no similar research has been conducted in Luxembourg the NCRE did not authorise us to contact neither the 242 patients who failed to respond, nor a family member.

### Instruments and their translation

As Luxembourg is multilingual and very culturally diverse (more than 170 different nationalities), our questionnaires were available in four languages: Luxembourgish, Portuguese, French and German. Most of the instruments were already available in French or English. The German, Portuguese and Luxembourgish versions were translated and back-translated, and proofread by native-speaking professional translators. As Luxembourg does not have academic medical facilities, all neurologists were trained elsewhere in Europe. The Luxembourg Society of Neurologists includes specialists who speak many languages and are culturally diverse. They collaborated in supervising the conception of all documents, the questionnaire for the patients and the caregivers, and their translation.

### Data collected from cerebrovascular disease patients

#### American Heart Association Stroke Outcome Classification: AHA.SOC

Stroke patient neurological impairments and residual disabilities were documented in motor, sensory, vision, cognition and language functions [29]. The AHA.SOC is a validated system that synthesises stroke-related phenomena in a single summary score. The number of deficiency domains affected was expressed in four categories: “no domain impaired”, “one domain impaired”, “two domains impaired, and “more than two domains impaired”.

#### Life satisfaction (dependent variable): LS

This is a single measure in which respondents self-rate their life satisfaction: “On a scale from 1 to 10, where would you place your level of satisfaction with your life?” (10 being the highest) [4].

#### Newcastle stroke-specific quality of life measure: Newsqol

This instrument consists of 11 subscales (Additional file 1: Appendix 1 [26]): mobility (Cronbach alpha α = 0.941), self-care (α = 0.930), pain/sensory (α = 0.649), cognition (α = 0.811), vision (α = 0.811), communication (α = 0.819, feelings (α = 0.874), interpersonal relationships (α = 0.657), emotion (α = 0.754), sleep (α = 0.824), and fatigue (α = 0.673). Newsqol responses as used here ranged from 1 (worst possible) to 4 (best possible). It is easy to administer, complete and score. The internal consistency reliability, content and discriminant validity were examined [27]. Scores for each dimension were calculated by summing the responses and then applying the formula (S-p)/(3 x p) x 100 (p = number of items in the dimension) to give scores ranging from 0 (worst QoL) to 100 (best QoL). Some items included a fifth response meaning that the individual was not affected by the issue or reflecting a state prior to the onset of the stroke. In this case, as the authors suggest, the response was assigned to the best possible QoL (representing ‘no impact of the stroke’).

#### Socioeconomic characteristics

The following socio-demographic characteristics were collected for all study participants: age, sex, nationality (Luxembourgish; others); educational level (under 12^th^ grade; 12^th^ grade and above), occupation at the time of the stroke onset (never employed; manual worker; employee/intermediate professional/technician; farmer; manager/professional), current occupational status (working; retired; unemployed), income (cut-off point of 36,000€; this represents three times the minimum wage in 2008). The municipalities were arranged in three groups: (Luxembourg City; 10 communes of more than 7,500 inhabitants; other municipalities) [30].

### Data collected from family caregivers

#### Life satisfaction (dependent variable) and socioeconomic characteristics

The same tools and procedure were used for the patients; in addition to the LS scale and socio-demographic data, information about the relationship with the care-recipient (i.e. spouse/ partner/other) was collected.

#### Short-form of the World Health Organization’s quality of life: Whoqol-bref

The Whoqol-bref allows for a detailed assessment of each individual aspect of QoL. It has four domains: physical health, psychological, social relationship and environment. To provide a broad and comprehensive assessment, one item is taken from each of the 24 facets from the four domains: seven are related to physical health, six to psychological status, three to social support and eight to the environment domain of the patient. Raw scores from the questionnaire are converted into the transformed score with the help of a Table, to give what is considered to be the final score in that particular domain. As no cut-points exist to categorise QoL measured by Whoqol-bref, the final score ranges from 0 to 100, with higher scores indicating better QoL. The subscales of the Whoqol-bref have been assessed [31]: physical (α = 0.763), psychological (α = 0.762), social relationships (α = 0.703), and environment (α = 0.785). The Whoqol-bref has been shown to be a multi-dimensional generic profile suitable for different cultural contexts among the general population [32] and informal caregivers [33]. It is a widely used tool with proven internal consistency reliability, content, discriminant validity [31]. It has been translated into the languages of this study: German [34], French [35] and Portuguese [36].

### Statistical analysis

For all measures the higher the score the better LS or QoL. The analyses were performed among stroke survivors and family caregivers. To assess the effects of patients’ socioeconomic factors and functional impairments on their LS a multiple regression model was used by retaining in the model only the factors associated with LS with p < 0.10. This model also included interaction terms between socioeconomic factors and functional impairments. A similar model was used to assess the effects of patients’ socioeconomic factors and functional impairments on the family caregivers’ LS. Finally, we evaluated the relationships between stroke survivors’ LS and patients’ QoL (measured with the 11 Newsqol dimensions) and family caregivers’ QoL (measured with the four Whoqol-bref domains) by using also multiple regression model which yield regression coefficient (standard error) adjusting for sex, occupational status, impaired motor function, and impaired memory functions. A similar model was used to evaluate the relationships between family caregivers’ LS and patients’ QoL and family caregivers’ QoL.

## Results

The participation rate was 94/374 = 25.1%. Comparisons between the socio-demographic characteristics (age, gender, nationality, commune of residence, number of admissions to hospital) of the study sample and of the general population showed no differences [28]. 

### Description of stroke survivors and family caregivers

Among the 94 patients (mean age 65.5 years), 32 had no caregivers (67.6 years), and 62 had a family caregiver (64.4 years). Mean LS of patients was 7.1/10, that of caregivers 7.2/10.

Table 1 shows that patients were mainly men, Luxembourgish, and were, at the time of the stroke onset, manual workers (32.5%) or intermediate professionals (31.3%). The majority were retired. Patients with caregivers had higher education and income than those with no caregivers. The neurological impairments affected a number of functions: sensory (44.7%), motor (35.1%), memory (31.9%), language (30.9%), and visual (20.2%). Impaired sensory and memory functions were more common among stroke survivors with caregivers than among the others (53.2% vs. 28.1%; 38.7% vs. 18.8%, respectively). The prevalence of two or more domains impaired was also higher among patients with family caregivers (43.5% vs. 21.9%).

**Table 1:** Characteristics of stroke patients and family caregivers

		**All stroke survivors N = 94**	**Stroke survivors without caregivers N = 32**	**Stroke survivors with caregivers N = 62**	**Family caregivers N = 62**
**Age**		65.5 (14.4)	67.6 (11.3)	64.4 (15.8)	59.3 (13.7)
**Life satisfaction [1;10]+**	Dependent variable	7.1 (1.9)	7.1 (2.3)	7.1 (1.6)	7.2 (1.7)
**Sex**	Women	44.7	53.1	40.3	65.6
	Men	55.3	46.9	59.7	34.4
**Nationality**	Luxembourgish	74.5	-	-	-
	Other	25.5	-	-	-
**Relationship with the patient**	Spouse / partner	-	-	-	82.3
	Child / Other	-	-	-	17.7
**Educational level**	Under 12^th^ grade	53.5	68.8	44.4	42.4
	12^th^ grade and above	46.5	31.2	55.6	57.6
**Occupation at the time of the stroke**^**1**^	Never employed	15.7	6.9	20.4	17.5
	Manual worker	32.5	51.7	22.2	14.0
	Employee / intermediate professional / technician	31.3	24.1	35.2	49.1
	Manager / Liberal Profession / Farmer	20.5	17.2	22.2	19.3
**Current occupational status**	Working	15.9	9.4	19.6	35.6
	At home without activity	27.3	34.4	23.2	30.5
	In retirement	56.8	56.3	57.1	33.9
**Income**^**2**^	< 36 000 €	46.4	70.4	31.0	28.9
	≥ 36 000 €	53.6	29.6	69.0	71.1
**Municipality**^**3**^	Luxembourg city	9.6	3.1	12.9	-
	Most populous municipalities	27.7	21.9	30.6	-
	Other municipalities	62.8	75.0	56.5	-
**Current AHA.SOC**^**4**^	Impaired motor	35.1	37.5	33.9	
**impairments functions**	Impaired visual	20.2	15.6	22.6	
	Impaired sensory	44.7	28.1	53.2	
	Impaired language	30.9	21.9	35.5	
	Impaired memory	31.9	18.8	38.7	
**Current AHA.SOC**^**4**^**domains**	0 domain impaired	28.7	40.6	22.6	
**neurological impairment**	1 domain impaired	20.2	25.0	17.7	
	2 domains impaired	14.9	12.5	16.1	
	more than two domains	36.2	21.9	43.5	

Mean (standard deviation) or %.

^1^ For unemployed and retired people as well as for those in vocational training at the time of the event, the last occupational activity was recorded.

^2^ Income value of reference [30].

^3^ The most populated are the communes of more than 7500 inhabitants (10 communes) [30].

^4^ American Heart Association Stroke Outcome Classification: AHA.SOC [29], stroke survivor neurological impairments and residual disabilities were documented in motor, sensory, vision, affect, cognition and language functions. This follows the AHA.SOC, a validated system that synthesises stroke-related impairments in a single summary score.

Based on extracted data from hospital medical records, the medical investigator had observed that the subtypes of the cerebrovascular disease represented 66.4% ischemic and 33.2% haemorrhagic. Arterial hypertension was the most frequent risk factor (80.8% vs. 75.3% haemorrhagic). More than 50% of the patients presented elocution troubles and nearly 80% had neurological deficits. Nearly 40% of the patients presented with a known dyslipidaemia, 22.7% suffered from diabetes, and 20.9% were obese [28].

Table 1 further shows that most family caregivers (mean age 59.3 years) were spouses, educated to the 12^th^ grade, and were, at the time of stroke, employees, technicians or intermediate professionals. Two years later, a third worked (35.6%), and 71.1% earned more than 36.000€ per year.

### Quality of life of stroke patients (Newsqol dimensions) and family caregivers (Whoqol-bref domains)

Table 2 reports that the less good Newsqol dimensions were emotion (mean 72.5/100), sleep (75.1), and cognition (76.3). All dimensions were higher for the 32 patients without caregivers, except the cognition which was lower for patients with caregivers (73.5 vs. 81.1). Regarding caregivers, the less good Whoqol-bref domains were psychological and environment ones (70.0 and 73.0, respectively).

**Table 2:** Quality of life scores of stroke survivors and family caregivers

		**All stroke patients N = 94**	**Patients Without caregivers N = 32**	**Patients with caregivers N = 62**	**Family caregivers N = 62**
**Stroke-patients’ quality of life**	Mobility	82.5 (23.2)	83.8 (20.3)	81.8 (24.8)	
**(Newsqol dimensions)**	Self-care	86.6 (22.6)	88.9 (20.2)	85.3 (23.8)	
	Pain	78.9 (27.4)	80.6 (27.7)	78.0 (27.5)	
	Cognition	76.3 (23.8)	73.5 (24.7)	81.1 (21.6)	
	Vision	85.0 (23.9)	86.5 (23.4)	84.2 (24.5)	
	Communication	81.3 (21.9)	87.0 (16.1)	78.1 (24.1)	
	Feelings	77.2 (24.8)	82.4 (25.6)	74.2 (24.0)	
	Interpersonal relationships	89.0 (15.4)	90.7 (18.1)	88.1 (13.8)	
	Emotion	72.5 (25.2)	77.9 (24.1)	69.5 (25.5)	
	Sleep	75.1 (23.9)	82.5 (22.0)	71.0 (24.2)	
	Fatigue	79.5 (24.6)	88.9 (18.1)	74.3 (26.2)	
**Family caregivers’ quality of life (Whoqol-bref domains)**	Physical health				75.4 (15.0)
	Psychological health				70.0 (17.0)
	Environment				73.0 (16.4)
	Social relationships				77.4 (13.9)

Mean (standard deviation).

### Associations of stroke survivors’ LS and family caregivers’ LS with patients’ socioeconomic factors and impaired motor and memory functions

Table 3 shows that among stroke patients, LS was higher for women (+12.4) and lower for patients at home with no occupation (−13.1, compared with retired people). Family caregiver’ LS was affected by caring for patients with impaired memory function (−12.8).

**Table 3:** Associations of stroke survivors’ life satisfaction (LS) and family caregivers’ LS with socioeconomic factors and impaired motor and memory functions: regression coefficients and 95% CI

		**Stroke patients’ LS**				**Family caregivers’ LS**			
		**Est.**	**IC 95%**		**p**	**Est.**	**IC 95%**		**p**
Intercept									
Sex	Female	12.40	1.67	22.85	**<0.03**	−8.18	−19.63	3.04	0.152
	Male	0.00				0.00			
Occupational status	Working	−7.00	−19.12	5.20	0.260	2.43	−9.13	13.66	0.661
	At home without activity	−13.07	−25.70	−0.63	**<0.05**	−3.37	−16.34	10.14	0.606
	At the retirement	0.00				0.00			
Impaired motor function	Yes	−7.47	−17.16	1.92	0.112	−2.95^(1)^	−13.89	8.14	0.600
	No	0.00				0.00			
Impaired memory function	Yes	−3.39	−12.50	5.78	0.469	−12.81^(2)^	−23.62	−1.96	**<0.02**
	No	0.00				0.00			

^1^ This term should be interpreted as the effect on the Caregiver’ LS of caring for a patient with impaired motor function.

^2^ This term should be interpreted as the effect on the Caregiver' LS of caring for a patient with impaired memory function.

### Associations of stroke survivors’ LS and family caregivers’ LS with patients’ Newsqol and family caregivers’ Whoqol-bref

As Table 4 shows, adjusted for sex, occupational status, and impaired motor and memory functions, patients’ LS was higher when scores increased for the Newsqol dimensions feelings (slopes 0.31), sleep (0.26), emotion (0.22), cognition (0.21) and pain (0.20), but did not correlate with any caregivers’ Whoqol-bref domain.

**Table 4:** Associations of stroke patients’ life satisfaction and family caregivers’ life satisfaction with stroke patients’ quality of life and family caregivers’ quality of life: slopes and 95%CI computed via multiple regression models with adjustment for sex, occupational status, and impaired motor and memory functions

	**Stroke patients’ life satisfaction**			**Family caregivers’ life satisfaction**		
	**Slope**	**CI 95%**	**P**	**Slope**	**CI 95%**	**P**
**Stroke-patients’ quality of life (Newsqol dimensions)**^**1**^**[0;100] +**						
Mobility	0.18	[−0.01;0.38]	0.062	0.11	[−0.10;0.31]	0.303
Self-care	0.11	[−0.09;0.31]	0.287	0.04	[−0.18;0.26]	0.686
Pain	0.20	[0.04;0.37]	**<0.05**	0.16	[−0.02;0.35]	0.088
Cognition	0.21	[0.03;0.38]	**<0.05**	0.20	[−0.02;0.41]	0.075
Vision	0.16	[−0.02;0.34]	0.085	0.06	[−0.16;0.28]	0.554
Communication	0.18	[−0.01;0.38]	0.066	0.08	[−0.14;0.29]	0.482
Feelings	0.31	[0.14;0.49]	**<0.000**	0.22	[0.01;0.42]	**<0.05**
Interpersonnal relationships	0.26	[−0.01;0.53]	0.063	0.28	[−0.08;0.63]	0.123
Emotion	0.22	[0.06;0.38]	**<0.01**	0.22	[0.03;0.41]	**<0.05**
Sleep	0.26	[0.08;0.45]	**<0.01**	0.10	[−0.11;0.31]	0.339
Fatigue	0.16	[−0.01;0.34]	0.064	0.15	[−0.05;0.34]	0.142
**Caregivers quality of life (Whoqol-bref domains)**^**1**^**[0;100] +**						
Physical health	0.20	[−0.17;0.54]	0.280	0.64	[0.31;0.97]	**<0.000**
Psychological health	0.10	[−0.22;0.41]	0.541	0.67	[0.37;0.97]	**<0.000**
Environment	0.12	[−0.18;0.41]	0.425	0.53	[0.22;0.83]	**<0.001**
Social Relationship	0.34	[−0.05;0.70]	0.086	0.68	[0.30;1.06]	**<0.001**

^1^Stroke caregivers (each dimension was analysed separately).

Family caregivers’ LS was associated with patients’ feelings and emotion dimensions (slopes 0.22 and 0.22, respectively). It was strongly linked with all caregivers’ Whoqol-bref domains: social relationships (slope 0.68), psychological health (0.67), physical health (0.64), and environment (0.53).

## Discussion

This study demonstrated that, two years after cerebrovascular disease onset, survivors’ life satisfaction was higher among women and lower among patients at home with no occupation (compared with retired people) and that family caregivers’ life satisfaction was affected by caring for patients with impaired memory function. It further showed that patients’ life satisfaction positively correlated with the five following Newsqol dimensions: feelings, sleep, emotion, cognition and pain, but did not correlate with any caregivers’ Whoqol-bref domain. Family caregivers’ life satisfaction was associated with patients’ feelings and emotion dimensions only. It was strongly linked with the four caregivers’ Whoqol-bref domains (physical health, psychological health, environment, and social relationships). Many patients suffered from impaired sensory function (44.7%), motor function (35.1%) and memory function (31.9%).

The main finding of our study at home is the similarity of associations of both patients’ LS and family caregivers’ LS with feelings and emotion stroke-related Newsqol dimensions. This suggests that strong psychological repercussions may be generated for both patients and family caregivers. If we intervene at the level of the determinants of QoL of patients, we would improve their satisfaction with regard to life. Using Newsqol revealed strong associations between four dimensions of pain, cognition, sleep and fatigue due to the stroke, and patients’ LS. This is of interest and not previously reported (in terms of stroke-specific QoL measures). Consequently, physical and psychological suffering as a repercussion of cerebrovascular disease needs to be assessed. The Newsqol appeared here as an appropriate tool to produce useful indicators to be considered in programs for stroke patients with disabilities [24]. Stroke-related upheavals can be minimised if professionals assist patients with a healthcare and social system which would provide preventive intervention with video, and psychosocial services using new IT technology (telephone, videophone, telestroke) [37].

Our study revealed relationships of patients’ LS with occupational status and gender which also deserve attention. Gender may include both gender social role and sex (biological meaning). We observed that patients with no occupational activity had lower LS. Having a job was linked with moderate LS and retired people had the best LS. We have no definitive explanation for these findings, but some hypotheses arise. First, unlike people at home with no activity and despite their handicap, retired patients may have less stress or unhappiness and may maintain a social position/identity, which is based not only on age and social characteristics but also on individual’s sense of self [38]. Secondly, access to medical aids and rehabilitation activities, as well as healthcare and prevention, may be easier as retired people have more spare time than working people. Thirdly, participation in community activities to promote ‘health capability’ (health functioning and ability to achieve health goals they value and act as agents of their own health) may be easier among retied people [39]. These activities may improve ability to make relevant choices to promote better health. It may also help to avoid resentment and promote psychological strength and confidence in the future [40]. The fact that the LS was higher in female than in male patients calls for further research on severity, symptoms and potential risk factors such as health-related behaviours, nutrition, leisure, etc. A recent review of literature about stroke risk factors and warning signs reported that, according to most studies, women know more about cerebrovascular disease than do men in most studies [41]. Women are more likely than men to report non-specific "somatic" symptoms and to report changes in their mental status [42]. Our findings are not consistent with those of the European LS survey [4], which reported small gender difference in various countries. However, this survey found that married people are more satisfied than those who are separated, divorced or widowed, and slightly more satisfied than single people in various country groups. In our dyads, most caregivers were women looking after spouses.

We found that caregivers of patients suffering from impaired memory had lower LS. This may reflect the repercussions of caring for patients with difficulties related to memory loss. Most neurological impairments concerned sensory and memory functions, but more survivors issued from our sample with a caregiver declared sensory and memory-related problems, and two or more domains impaired. Family caregivers had to cope with physical disabilities, but also psychological problems, making some of them exhausted. Home-based rehabilitation requires stroke patients and their caregivers to find new ways, within their families, to solve problems, communicate and deal emotionally with others. Individual or community interventions should redefine their resulting needs [43]. Our result brings to light some contrasting aspects of the literature which suggest that despite the socioeconomic differences between care settings*,* cerebrovascular disease is a life-threatening and potentially disabling event as well as an important family issue, particularly for spouses [44]. Under Swedish law, the repercussions for informal caregivers have been recognised by an amendment in 2009 which calls for assistance in order to minimize the physical and psychological strain on caregivers and caregiving burden. It also recognises the caregivers’ perspective in guiding interventions, such as support groups and home help. This implies that, within rehabilitation, the dyad could be viewed as one client, with the potential of benefitting from support [45].

We further found that, in accord with previous studies [7,12,15], LS was associated with the QoL among family caregivers. All QoL domains were involved: physical health, psychological health, living environment, and social relationship. In contrast, to that, we failed finding an association between stroke survivors’ LS and caregivers’ QoL. This result may be a bit surprising, but it supports the hypothesis that caregivers may not leave caregiving related to their own health-related issues. It may be noted that the LS of study patients and caregivers (7.1 and 7.2/10, respectively) was lower than the national LS indicator in Luxembourg (in 2007, 7.85/10), which was higher than that from the European Quality of Life Survey [4] (7.0 for EU-27), but behind Denmark, Finland and Sweden.

Contextualising our findings poses a challenge for a number of reasons, in particular the economic situation (as regards Luxembourg’s gross domestic product per inhabitant), and the fact that Luxembourg is one of the smallest European countries (524.853 inhabitants (January 2012) area 2600 km^2^ ) and small distances between the population and the health systems. The care structures are thus geographically accessible for the whole population. The socio-demographic characteristics of the study patients (53.6% had an income of more than 36,000€ and 74.5% were of Luxembourgish nationality) also suggest that social and medical supports focussed on community professional-oriented services are easily available. Difficulties associated with maintaining inner-city medical practices [46] and community-care provision vary substantially according to location and income. These factors also influence domiciliary care delivery: distribution of resources at local levels; financial constraints; and the application of eligibility criteria in providing medical and community services [47].

### Strengths and limitations

Among stroke patients, aphasic patients were also included in the study sample with appropriate training for researcher to communicate with them and specific interview protocol. As in the European survey the LS assessment was based on one question easy to routinely use. QoL was measured using two internationally accepted instruments: on the one hand, the Newsqol to measure specific issues of patients; and on the other hand, the Whoqol which is a good transcultural instrument appropriate for caregivers. Studying two-year post cerebrovascular disease is an opportunity to provide valuable information on patient-caregiver monitoring over time. Certain pathology may mimic stroke [48]. In addition, by two years post-stroke patients and their family caregivers may have adapted to their new situation, reorganised their daily lives, and become accustomed to caregiving [12]. Such a study protocol remains rare because it is very expensive, and it is not easy to organise a study 24 months after stroke onset. The participation rate is rather small but similar to recent literature (27%) [45]. Certain patients died, lived in institutions, changed their residence (for example with their son or daughter), or failed to respond.

### Practical implications

Our study highlights that LS and QoL assessments are simple and may be used to identify cerebrovascular disease patients at risk and adverse health issues that may be targets for interventions in order to sustain long-term hospital discharged medical care. Home-based rehabilitation would improve in sustainability if patients and caregivers could benefit from a follow-up with assessment of medical, material, psychosocial, and information needs [3]. A telephone service by trained health and social professionals may help. Telestroke (for example with videoconferencing) may reduce stress, provide reassurance about secondary treatment effects, improve compliance with prescriptions, and give information about medical-social services [37]. Stroke-patients and family caregivers who participate in a problem-solving intervention group (face-to-face, family-adapted training at the caregiver's home and telephone counseling) improve their problem-solving skills and caregiving preparedness; they report better vitality, social functioning, mental health, and role limitations related to emotional problems [49,50]. Appropriate information may promote autonomy and decision ability. Patient-centred care needs effective collaboration between various professionals (psychologists, nurses, social workers, general practitioners, neurologists, etc.), patients, and their families. Our findings may be useful for policy makers to understand the family and medical-social contexts of stroke over time in order to design adequate health systems [51,52].

## Conclusions

Two years after stroke onset, a great proportion of cerebrovascular disease patients suffered from impaired sensory function (44.7%), motor function (35.1%) and memory function (31.9%). Life satisfaction was better among women and worse among unemployed socioeconomically active patients. Among survivors, life satisfaction was worse in those suffering from feeling, sleep, emotion, cognition and pain issues, but did not correlate with caregivers’ quality of life. Among family caregivers, life satisfaction was worse in those with patients suffering from issues of feeling (less independent, yourself, life changed, depressed, useless, less control because of stroke) and emotion (get more emotional, fear of another stroke or to become dependent on others); it was associated with all caregivers’ Whoqol-bref domains (physical health, psychological health, environment, and social relationships).The life satisfaction measure, Newsqol, and Whoqol appeared to be good appropriate tools. Our findings may be useful for policy makers about family and medical-social issues of stroke home-based rehabilitation over time.

## Competing interests

The authors declare that they have no competing interests.

## Authors’ contributions

MB conceived and carried out the research; she was the scientific director of the study and had main responsibility for writing the manuscript. SC conceived the protocol as medical investigator and analysed the data. ELB realised the statistical analysis and participated in data analysis. NC participated in data analysis and in writing the manuscript. All authors read and approved the final manuscript.

## Pre-publication history

The pre-publication history for this paper can be accessed here:

http://www.biomedcentral.com/1471-2377/12/105/prepub

## Supplementary Material

Additional file 1 **Appendix 1.** Newcastle stroke-specific quality of life measure (Newsqol) by Buck et al. [26]. Click here for file
