# Supplementary material for: Life satisfaction two-years after stroke onset: the effects of gender, sex occupational status, memory function and quality of life among stroke patients (Newsqol) and their family caregivers (Whoqol-bref) in Luxembourg
Source: BMC Neurol. 2012 Sep 25;12:105. doi: 10.1186/1471-2377-12-105 (PMC3551740; doi:10.1186/1471-2377-12-105)
Supplement: Additional file 1 — Appendix 1. Newcastle stroke-specific quality of life measure (Newsqol) by Buck et al. [26]. [file 1471-2377-12-105-S1.doc]

| **Mobility** | |
| --- | --- |
| 1. | Do you get around in a wheelchair because of the stroke? |
| 2. | Because of the stroke, do you have any difficulty walking half a mile? |
| 3. | Because of the stroke, do you have any difficulty walking up or down highlife satisfaction? |
| 4. | Because of the stroke, do you walk with a stick or frame or by holding onto things? |
| 5. | Do you feel as if you walk slowly because of the stroke? |
| 6. | Do you have difficulty managing stairs on your own because of the stroke? |
| 7. | Do you have difficulty bending down because of the stroke? |
| 8. | Do you find that you are unsteady on your feet because of the stroke? |
| 9. | Because of the stroke, do you have difficulty standing for any length of time? |
| **Self-care** | |
| 10. | Do you have difficulty with housework because of the stroke? |
| 11. | Because of the stroke, do you have difficulty with cooking? |
| 12. | Because of the stroke, do you have difficulty preparing food, for example cutting a slice of bread or cutting up vegetables? |
| 13. | Do you have difficulty managing the shopping because of the stroke? |
| 14. | Because of the stroke, do you have difficulty using public transport? |
| 15. | Do you have difficulty getting washed by yourself because of the stroke? |
| 16. | Because of the stroke, do you have difficulty getting dressed by yourself, including things like zips and buttons? |
| 17. | Do you have difficulty getting in/out of the bath or shower on your own because of the stroke? |
| **Pain / sensory** | |
| 18. | Do you have any pain because of the stroke? |
| 19. | How often do you have pain because of the stroke? |
| 20. | Because of the stroke, do you have difficulty picking up small things? |
| **Vision** | |
| 21. | Do you have problems with your eyesight because of the stroke? |
| 22. | Do you have any difficulty with reading because of your eyesight (because of the stroke)? |
| **Cognition** | |
| 25. | Do you find it difficult to concentrate for long because of the stroke? |
| 24. | Because of the stroke, are there times when you forget what you have said or what people say to you? |
| 23. | Because of the stroke do you find it difficult to solve problems or make decisions? |
| 26. | Would you say you keep forgetting things because of the stroke? |
| 27. | Because of the stroke, do you find it difficult to think clearly? |
| **Communication** | |
| 28. | Do you feel as though your speech is slurred at all because of the stroke? |
| 29. | Do you find it difficult to make yourself understood because of the stroke? |
| 30. | Because of the stroke, are there times when you have difficulty expressing yourself? |
| 31. | Do you have any difficulty with writing because of the stroke? |
| **Feelings** | |
| 32. | Do you feel less independent than you were, because of the stroke? |
| 33. | Has the stroke changed the way you feel about yourself? |
| 34. | To what extent would you say your life has changed because of the stroke? |
| 35. | Do you feel depressed because of the stroke? |
| 36. | Does the stroke make you feel useless at all? |
| 37. | Do you feel you have less control over what is happening in your life because of the stroke? |
| **Interpersonal relationships** | |
| 38. | Because of the stroke, do you argue more with close friends or family? |
| 39. | Has the stroke put any strain on your relationship with your spouse or partner? |
| 40. | Does the stroke interfere with your sex life and if so, how much? |
| 41. | Are you short-tempered because of the stroke? |
| 42. | Are you less tolerant because of the stroke? |
| 43. | Because of the stroke, have you become nervous about meeting people? |
| **Emotion** | |
| 44. | Do you get more emotional because of the stroke? |
| 45. | Do you sometimes cry at the least thing because of the stroke? |
| 46. | Are you worried that you could have another stroke? |
| 47. | Because of the stroke, do you worry about becoming dependent on other people? |
| **Sleep** | |
| 48. | Do you have problems sleeping at night because of the stroke? |
| 49. | Do you have difficulty getting off to sleep because of the stroke? |
| 50. | Because of the stroke, do you sometimes wake up too early? |
| 51. | Do you find you need a lot of rest because of the stroke? |
| 52. | Do you ever feel exhausted because of the stroke? |
| 53. | Do you feel that you lack energy because of the stroke? |
| **Fatigue** | |
| 54. | Because of the stroke, are there days when you feel you could sleep all the time? |
| 55. | Do you doze off during the day because of the stroke? |
| 56. | Because of the stroke, do you feel that you can't be bothered with things at times? |
